# Zero‐Fluoroscopy Radiofrequency Ablation of a Right Ventricular Outflow Tract PVC in a Patient With Situs Inversus Totalis: When Precision Matters

**DOI:** 10.1155/cric/1241820

**Published:** 2026-04-15

**Authors:** Eduardo Sanhueza, Matthew Hanson, Victor Neira, Sanoj Chacko, Andres Enriquez, Roji Oli, Mariana Saramago, Cengiz Burak

**Affiliations:** ^1^ Division of Cardiology, Queen’s University, Kingston, Ontario, Canada, queensu.ca; ^2^ Division of Cardiology, University of Pennsylvania, Philadelphia, Pennsylvania, USA, upenn.edu

**Keywords:** cardiac ablation, dextrocardia, Kartagener syndrome, premature ventricular complex, situs inversus, zero fluoroscopy

## Abstract

**Background:**

Congenital cardiac anomalies present unique challenges for adult cardiac electrophysiologists, particularly when attempting complex ablation procedures.

**Case Summary:**

We report the case of a 60‐year‐old male with situs inversus totalis who presented with symptomatic, high‐burden premature ventricular complexes (PVCs) refractory to medical therapy. Using reversed (mirror‐image) precordial lead placement, high‐definition 3D electroanatomic mapping, and intracardiac echocardiography, successful radiofrequency ablation was achieved using a completely zero‐fluoroscopy approach.

**Discussion:**

In patients with congenital cardiac anomalies, the applicability of zero‐fluoroscopy strategies is often limited because of their complex anatomy. Successful management requires in‐depth anatomical understanding and carefully adapted procedural techniques. This case highlights that even in the setting of significant anatomical variation, a systematic zero‐fluoroscopy approach can lead to effective and safe outcomes.

**Take‐Home Message:**

Zero‐fluoroscopy PVC radiofrequency ablation using high‐definition 3D mapping and intracardiac echocardiography is a feasible and safe approach, even in anatomically challenging cases such as situs inversus.

Highlights

• The number of adult patients with congenital heart disease is steadily increasing. Electrophysiology teams must be prepared to manage a wide range of atrial and ventricular arrhythmias in this population, using tailored, safe, and effective approaches.
• Zero‐fluoroscopy radiofrequency ablation guided by high‐definition 3D electroanatomic mapping and intracardiac echocardiography (ICE) is feasible and safe, even in challenging cases such as situs inversus totalis, offering a precise, effective, and radiation‐free approach for managing arrhythmias.

## 1. History of Presentation

A 60‐year‐old man with a history of situs inversus totalis presented with a 4‐year history of recurrent palpitations, dizziness, and fatigue. Over the previous 8 months, these symptoms had intensified, significantly impacting his quality of life. Holter monitoring documented a high burden of monomorphic premature ventricular complexes (PVCs) of 22%. Treatment with bisoprolol followed by sotalol provided limited benefit and was poorly tolerated.

## 2. Past Medical History

**Figure 1:**
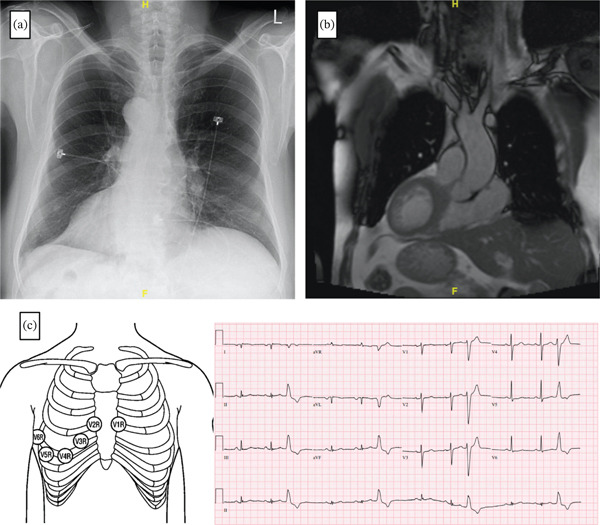
(a) Anteroposterior chest X‐ray showing dextrocardia, identified by the heart located on the right side of the thorax with the apex pointing to the right. This distinguishes dextrocardia from dextroversion, in which the heart is right‐sided, but the apex remains leftward. (b) Cardiac and abdominal magnetic resonance images demonstrating dextrocardia with situs inversus, evidenced by the mirror‐image arrangement of abdominal organs, including the liver, stomach, and spleen. (c) Diagram illustrating reversed (mirror‐image) precordial lead placement, along with a corresponding 12‐lead ECG acquired with mirrored precordial leads. The ECG displays a PVC with an inferior axis and left bundle branch block (LBBB) morphology and an R‐wave transition in Lead V5—findings consistent with a right ventricular outflow tract (RVOT) origin.

The patient has a diagnosis of situs inversus totalis with chronic sinusitis and bronchiectasis, consistent with Kartagener syndrome, a rare autosomal recessive ciliary disorder. Comprehensive cardiac imaging—including cardiac computed tomography (CT), cardiac magnetic resonance imaging (MRI), and coronary angiography—confirmed the presence of structurally normal cardiac anatomy in mirror‐image orientation, with no additional comorbidities identified (Figure 1A,B).

## 3. Management

An electrophysiology study was performed under conscious sedation. Precordial leads were placed in a mirror image (reverse configuration) on the right side of the chest. Due to the patient’s dextrocardia, defibrillator patches were also placed in a reversed configuration. The patient had rare PVCs at baseline, but the frequency increased with intravenous isoproterenol at 2 *μ*g/min. The predominant PVC morphology exhibited a left bundle branch block (LBBB) pattern, precordial transition at V3–V4, and inferior axis (Figure 1C). A second less frequent PVC had a morphology consistent with an origin from the moderator band.

**Figure 2:**
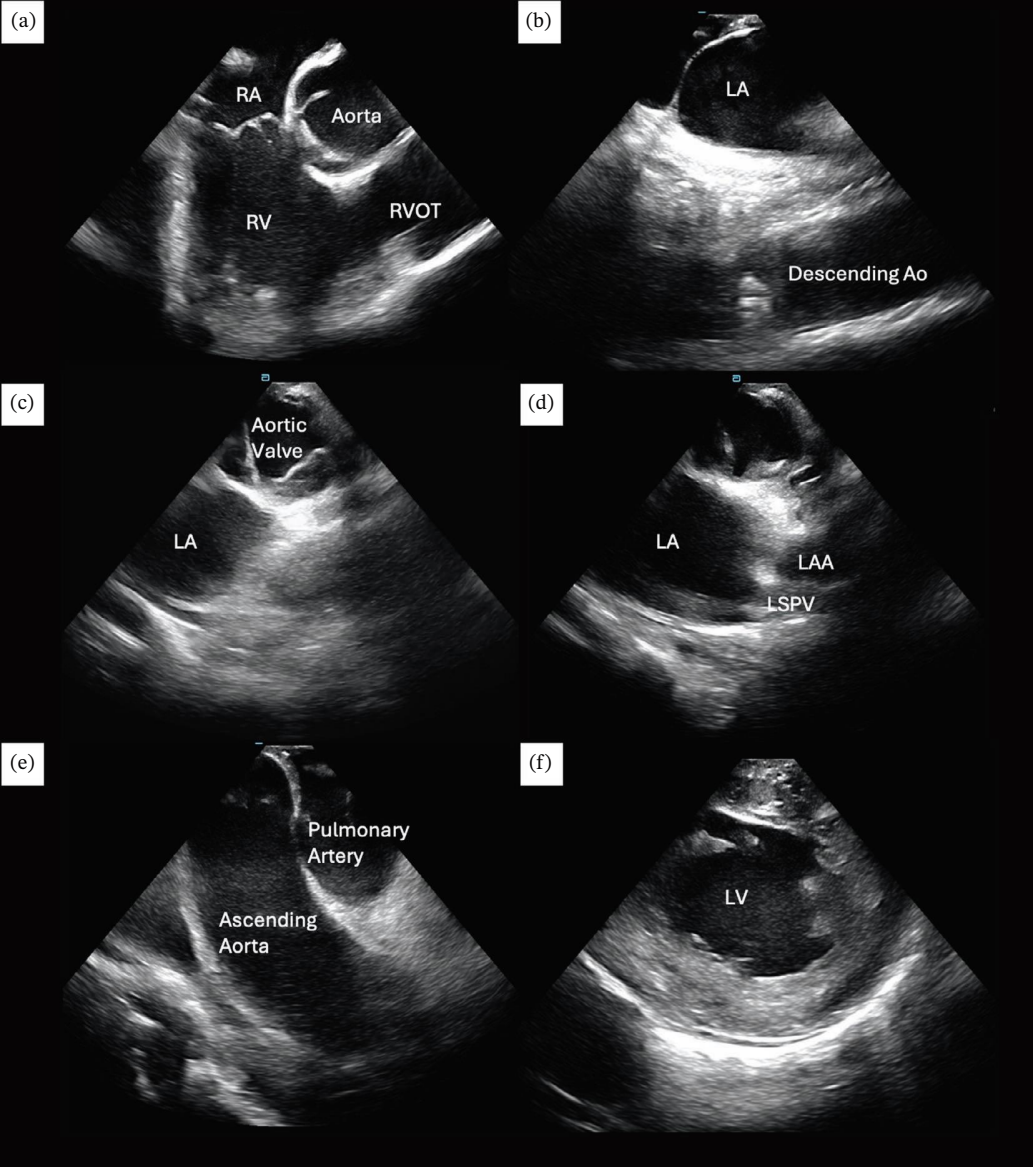
Intracardiac echocardiography of a patient with dextrocardia. (a) Home view. (b) Interatrial septum and descending aorta. (c) Aortic valve in short‐axis view. (d) Left atrial appendage and left superior pulmonary vein. (e) Ascending aorta and pulmonary artery. (f) Left ventricle. Abbreviations: LA, left atrium; LAA, left atrial appendage; LSPV, left superior pulmonary vein; LV, left ventricle; RA, right atrium; RV, right ventricle; RVOT, right ventricular outflow tract.

Electroanatomic mapping was performed using the EnSite X system (Abbott, St. Paul, Minnesota) in combination with the Advisor HD Grid mapping catheter (Abbott). ICE with a ViewFlex Xtra catheter (Abbott) provided real‐time anatomical visualization and guided catheter navigation. ICE images were obtained using standard maneuvers in reverse, beginning from the home view with a counterclockwise rotation (Figure 2). A decapolar catheter was positioned in the coronary sinus for anatomical reference, and a large deflectable sheath (Agilis, Abbott) was employed to optimize catheter maneuverability and stability.

**Figure 3:**
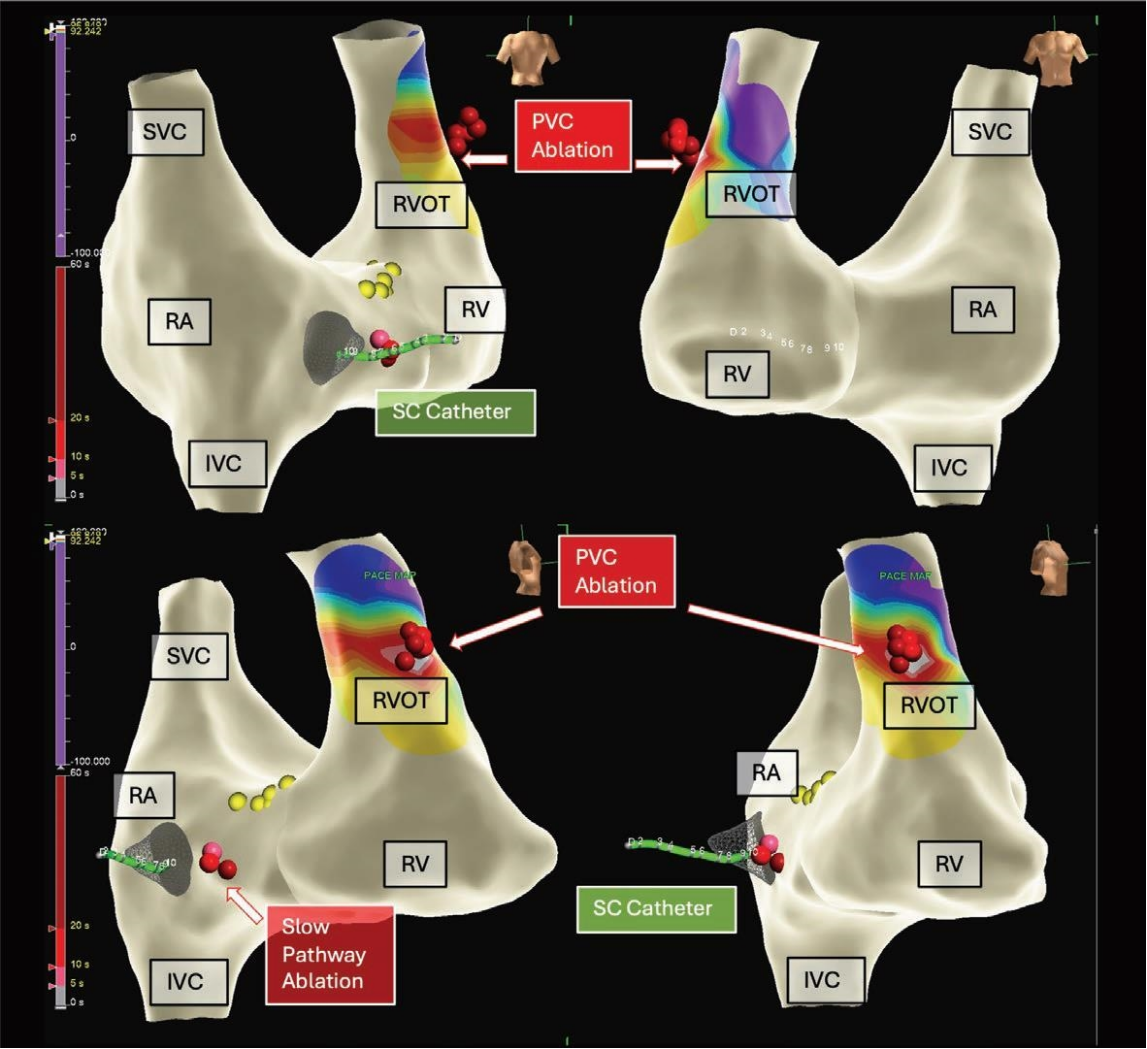
Three‐dimensional electroanatomic reconstruction with local activation time (LAT) mapping demonstrating the earliest activation at the anterolateral aspect of the right ventricular outflow tract (RVOT). Red dots denote radiofrequency (RF) ablation sites in the RVOT (anterolateral) and in the slow pathway region. Yellow dots indicate His bundle electrograms. Abbreviations: CS, coronary sinus; IVC, inferior vena cava; RA, right atrium; RV, right ventricle; SVC, superior vena cava.

**Figure 4:**
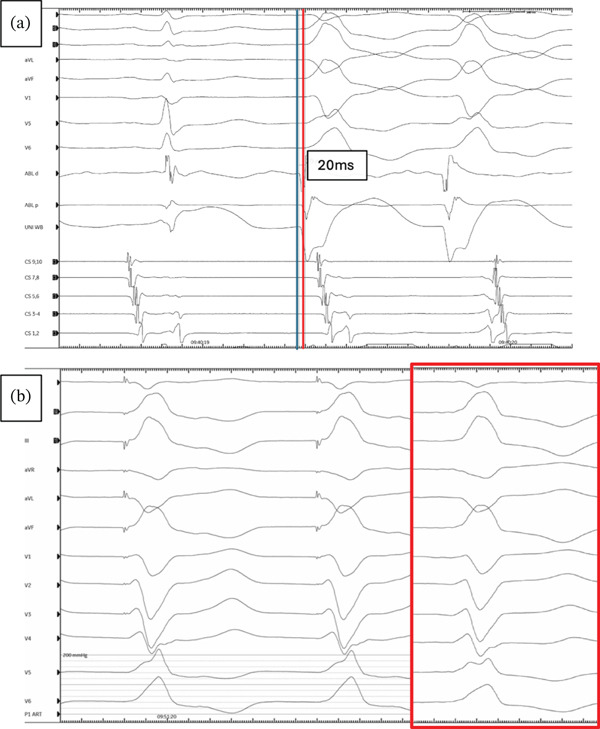
(a) Intracardiac electrograms showing early activation (20 ms pre‐QRS) at the anterolateral right ventricular outflow tract (RVOT). (b) Pace mapping at this site demonstrated a 97% match with the clinical PVC morphology (basal PVC template shown in red).

Activation mapping confirmed a septal right ventricular outflow tract (RVOT) origin, with local activation preceding the QRS onset by 20 ms and a 97% pace‐map match (Figures 3 and 4). Radiofrequency ablation was delivered at this site using an irrigated, sensor‐enabled catheter (TactiFlex, Abbott) at 30–35 W for 60–90 s, resulting in PVC suppression. Additional consolidation lesions were subsequently applied.

Following ablation, programmed ventricular stimulation and repeat isoproterenol infusion failed to induce PVCs. However, typical atrioventricular nodal reentrant tachycardia (AVNRT) was inducible, and dual AV nodal physiology was demonstrated. A slow pathway modification was performed, and no further inducible AVNRT or PVCs were observed.

## 4. Outcomes and Follow‐Up

The patient was monitored and discharged the day after the procedure without complications. At 6‐month follow‐up, he remained completely asymptomatic; Holter monitoring showed no PVCs.

## 5. Discussion

PVCs are common arrhythmias that may occur in structurally normal hearts or in the setting of underlying cardiomyopathy. In patients with a high PVC burden, symptoms such as palpitations, fatigue, and dizziness can significantly affect the quality of life. Moreover, frequent ectopy may result in PVC‐induced cardiomyopathy, characterized by a reduction in left ventricular ejection fraction [1].

Catheter ablation has emerged as a safe and effective therapeutic option for symptomatic or high‐burden PVCs, particularly those arising from the RVOT, with reported success rates of up to 80%–92% [2]. The efficacy of this approach depends in part on the precise localization of the ectopic focus. In this regard, 3D electroanatomic mapping systems have become indispensable, enhancing procedural accuracy and safety while minimizing the need for fluoroscopy [2].

Patients with congenital cardiac anomalies pose unique challenges for catheter ablation. The mirror‐image orientation of cardiac structures, absence of conventional anatomical landmarks, altered anatomy, and limited operator experience complicate the interpretation of electrograms and fluoroscopic images. However, with a growing number of adult patients living with congenital heart disease, familiarity with these scenarios is increasingly important in contemporary electrophysiology practice [3].

Dextrocardia is a rare congenital condition in which the heart is located in the right hemithorax with its apex pointing to the right. It occurs in approximately 1 in 20,000 live births and may present in isolation or in association with other congenital anomalies, including situs inversus and structural heart defects. There are three major types of “situs”: situs solitus (normal visceral arrangement), situs inversus (mirror image of normal anatomy), and situs ambiguous (also known as visceral heterotaxy or isomerism). About one‐third of patients with dextrocardia have complete situs inversus [4]. Kartagener syndrome is a rare autosomal recessive condition characterized by the triad of situs inversus, primary ciliary dyskinesia, and chronic respiratory tract infections, including bronchiectasis and nasal polyposis. Its prevalence is estimated at approximately 1 in 30,000 individuals [5]. In general, patients with dextrocardia and situs inversus without structural defects have a favorable prognosis and do not typically require surgical correction.

Several reports have documented successful catheter ablation in patients with dextrocardia. In 2014, Bonnemeier et al. [6] reported the first successful case of PVC ablation in a patient with dextrocardia, performed using fluoroscopy and pace mapping. In 2015, two different case reports described PVC ablation in patients with dextrocardia and situs inversus, utilizing reverse precordial lead placement and a 3D mapping system. Their findings demonstrated that mirror‐image ECG interpretation enabled the application of standard PVC localization algorithms [7]. In 2017, Chen et al. [8] reported the successful ablation of a left ventricular outflow tract PVC in a patient with dextrocardia and situs inversus, employing a combination of 3D mapping, cardiac CT imaging, and fluoroscopy.

Although reports of atrial fibrillation ablation in patients with dextrocardia are increasingly common [9], data on ablation of PVCs and ventricular tachycardia remain limited. These procedures require specific technical adaptations, including reverse ECG lead placement, high‐resolution electroanatomic mapping, and multimodal imaging—such as cardiac CT or MRI—for accurate delineation of the arrhythmic substrate. A critical consideration during electrophysiological procedures is the recognition of mirror‐image anatomy, in which standard spatial orientations are reversed: left becomes right, and clockwise rotations become counterclockwise [10].

In device procedures involving dextrocardia, horizontally flipped fluoroscopy images are often used to obtain operator‐familiar projections, like standard projections in levocardia [11]. However, in complex arrhythmia ablations, such strategies can alter tactile feedback and hand–eye coordination, potentially increasing procedural risk—particularly if the 3D mapping is not appropriately flipped. In our experience, real‐time ICE guidance combined with high‐definition mapping can help mitigate these challenges, allowing for precise catheter navigation and safe completion of the procedure.

This case demonstrates a successful zero‐fluoroscopy ablation of PVCs in a patient with situs inversus totalis, achieved through the combination of high‐density mapping and ICE guidance. It emphasizes the importance of tailoring procedural strategies to the individual patient and illustrates how modern mapping technologies can facilitate safe and effective arrhythmia management in patients with congenital cardiac variants. Importantly, by entirely avoiding radiation exposure, this approach is consistent with current guideline recommendations to minimize fluoroscopy use, providing benefits for younger patients or those who may require longer and repeated interventions.

## 6. Conclusion

In patients with mirror‐image anatomy such as situs inversus totalis, zero‐fluoroscopy catheter ablation of RVOT PVCs can be performed safely and effectively using high‐definition mapping systems and ICE. This case highlights the importance of individualized procedural planning and the utility of modern EP technologies in challenging anatomical contexts.

**Nomenclature**
PVCs: premature ventricular complexes
ICE: intracardiac echocardiography
RFA: radiofrequency ablation
CT: computed tomography
LBBB: left bundle branch block
RVOT: right ventricular outflow tract

## Funding

No funding was received for this manuscript.

## Ethics Statement

Written informed consent was obtained, and the Queen’s University Research Ethics Office exempted this case report from formal REB review (TCPS 2, Article 2.5).

## Conflicts of Interest

The authors declare no conflicts of interest.

## Data Availability

The data that support the findings of this study are available on request from the corresponding author. The data are not publicly available due to privacy or ethical restrictions.
